# Supplementary material for: Variability in ‘Capri’ Everbearing Strawberry Quality during a Harvest Season
Source: Foods. 2023 Mar 22;12(6):1349. doi: 10.3390/foods12061349 (PMC10048161; doi:10.3390/foods12061349)
Supplement: Supplementary file 1 [file foods-12-01349-s001.zip › foods-2206848-SI.pdf]

**Supplementary Material: Figure S1. Sorting of strawberries at harvest**

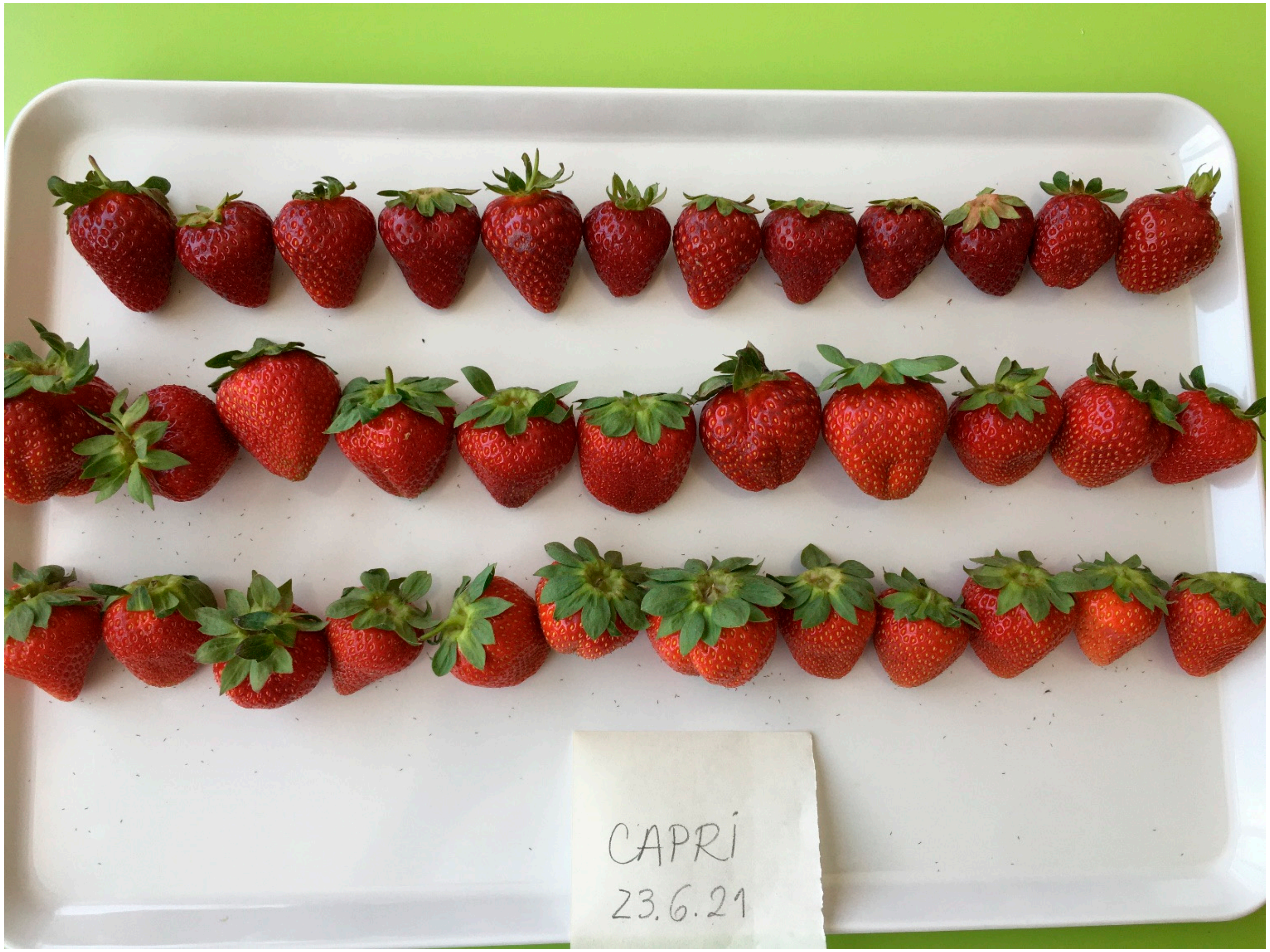

**Supplementary Material: Table S1. Identification of phenolic compounds and the standards used to express them**

| | *Positive mode $[M]^+$* | | | | | | |
|---|---|---|---|---|---|---|---|
| Peak No | Anthocyanin | Rt (min) | λ [nm] | $[M]^+$ (m/z) | $MS^2$ (m/z) | Expressed as | Group |
| 1A | cyanidin 3-*O*-glucoside | 9.91 | 530 | 449 | 287 | Cyanidin-3-O-glucoside | Anthocyanins |
| 2A | pelargonidin 3-*O*-glucoside | 11.20 | 530 | 433 | 271 | Pelargonidin-3-O-glucoside | Anthocyanins |
| 3A | pelargonidin 3-*O*-rutinoside | 11.60 | 530 | 579 | 271, 433 | Pelargonidin-3-O-glucoside | Anthocyanins |
| 4A | pelargonidin-3-(6″malonyl)glucoside | 15.61 | 530 | 519 | 271, 475, 433 | Pelargonidin-3-O-glucoside | Anthocyanins |
| 5A | 5-pyranopelargonidin-3-glucoside | 16.13 | 530 | 501 | 339 | Pelargonidin-3-O-glucoside | Anthocyanins |
| 6A | pelargonidin-3-*O*-acetylglucoside | 16.71 | 530 | 475 | 271 | Pelargonidin-3-O-glucoside | Anthocyanins |

| | *Negative mode $[M^- H]^-$* | | | | | | | | |
|---|---|---|---|---|---|---|---|---|---|
| Peak No | Phenolic compound | Rt (min) | λ [nm] | $[M-H]^-$ (m/z) | $MS^2$ (m/z) | $MS^3$ (m/z) | $MS^4$ (m/z) | Expressed as | Group |
| 1 | *p*-coumaric hexoside | 13.08 | 280, 350 | 325 | 163,145, 119 | | | *p*-coumaric acid | Hydroxycinnamic acid derivatives |
| 2 | *p*-coumaric hexoside der. | 13.60 | 280, 350 | 371 | 325 | 163, 145, 119 | | *p*-coumaric acid | Hydroxycinnamic acid derivatives |
| 3 | 1-*O*-feruoylglucose | 14.06 | 350 | 355 | 193, 217, 175 | | | ferulic acid | Hydroxycinnamic acid derivatives |
| 4 | propelargonidin dimer | 15.10 | 280 | 561 | 543, 435,289 | | | procyanidin B1 | Flavanol |
| 5 | brevifolin carboxylic acid | 15.33 | 280, 350 | 291 | 247 | 219, 191, 203, 175 | 191 | ellagic acid | Hydroxybenzoic acid derivatives |
| 6 | ferulic acid hexoside der. | 16.14 | 280, 350 | 499 | 455, 293 | 265, 264, 237 | 237 | ferulic acid | Hydroxycinnamic acid derivatives |
| 7 | apigenin-7-*O*-glucoside | 16.58 | 280 | 431 | 269 | 147, 241, 225 | | apigenin-7-glucoside | Flavonols |
| 8 | ferulic acid hexoside der. | 16.80 | 280, 350 | 449 | 355, 269, 193 | | | ferulic acid | Hydroxycinnamic acid derivatives |
| 9 | ellagic acid der. | 17.99 | 280 | 479 | 301, 300, 433 | 257, 229, 185 | | ellagic acid | Hydroxybenzoic acid derivatives |
| 10 | galloyl-bis-HHDP-glucose | 18.40 | 280 | 935 | 633, 301 | 257, 229, 185, 284 | | ellagic acid | Hydroxybenzoic acid derivatives |
| 12 | ellagic acid-*O*-deoxyhexoside | 19.33 | 280, 350 | 447 | 301, 300 | 257, 229 | | ellagic acid | Hydroxybenzoic acid derivatives |
| 13 | tormentic acid | 19.63 | 280 | 487 | 441, 339, 293 | 293, 149, 147 | 191, 101 | ellagic acid | not phenolic compound |
| 14 | ellagic acid-*O*-deoxyhexoside | 19.85 | 350 | 447 | 301, 300 | 257, 229 | | ellagic acid | Hydroxybenzoic acid derivatives |
| 15 | cinnamic acid-3-*O*-hexoside | 21.09 | 280, 350 | 355 | 309, 147, 207, 248 | | | caffeic acid | Hydroxycinnamic acid derivatives |
| 16 | isoquercetin | 21.35 | 350 | 463 | 301, 300 | 179, 151 | | quercetin-3-glucoside | Flavonols |
| 17 | *trans*-tiliroside | 21.75 | 350 | 593 | 285 | 257, 267, 241, 229, 213, 197 | | kaempferol-3-glucoside | Flavonols |
| 18 | quercetin-3-*O*-glucuronide | 22.26 | 280, 350 | 477 | 301 | 179, 151 | | quercetin-3-glucoside | Flavonols |
| 19 | kaempferol-*O*-hexoside | 22.70 | 350 | 447 | 284, 285 | | | kaempferol-3-glucoside | Flavonols |
| 20 | quercetin der. | 22.98 | 280 | 619 | 499, 521 | 301, 323 | 179, 151 | quercetin-3-glucoside | Flavonols |
| 21 | kaempferol-3-*O*-glucuronide | 23.51 | 280, 350 | 461 | 285 | 257, 267, 241 | | kaempferol-3-glucoside | Flavonols |
| 22 | caffeoylglucaric isomer der. | 24.26 | 280 | 417 | 371 | | | caffeic acid | Hydroxycinnamic acid derivatives |
| 23 | isorhamnetin-*O*-hexoside der. | 24.53 | 350 | 477 | 315, 379 | | | isorhamnetin-3-glucoside | Flavonols |
| 24 | kaempferol-*O*-acetylhexoside | 25.20 | 350 | 489 | 285 | 257, 267, 241 | | kaempferol-3-glucoside | Flavonols |

Rt, retention time; der., derivative; HHDP, hexahydroxydiphenoyl; $[M-H]^-$, pseudo-molecular ion identified in negative ion mode; $[M]^+$, pseudo-molecular ion identified in positive ion mode ion.
